# Mental health consultations during the perimenopausal age range: a qualitative study of GP and patient experiences

**DOI:** 10.3399/BJGP.2025.0069

**Published:** 2025-10-07

**Authors:** Jo Burgin, Yvette Pyne, Anna Davies, David Kessler

**Affiliations:** 1 Bristol Medical School, University of Bristol, Bristol, UK

**Keywords:** menopause, mental health, women’s health

## Abstract

**Background:**

There is an increased risk of mood changes in perimenopause, and evidence that patients and GPs may overlook this association. Evidence also shows that GPs have a lack of confidence in managing perimenopausal symptoms.

**Aim:**

To examine clinical consultations for patients in the perimenopausal age range who were presenting with mental health symptoms, and the experiences of GPs providing care to such patients.

**Design & setting:**

A qualitative study was undertaken in one integrated care system in south-west England. It involved 18 women aged 45–55 years, who had consulted with their GP about a mental health symptom in the previous 6 months, and 11 GPs.

**Method:**

Participants were recruited between February 2023 and August 2023. Data were collected through semi-structured interviews, and thematic analysis was used to identify recurring patterns and key insights regarding consultation practices, patient–GP communication, and gaps in education and training.

**Results:**

Women either did not recognise perimenopause or were uncertain whether it contributed to their mental health symptoms, and felt inhibited or embarrassed about raising the issue. GPs reported using variable approaches to asking about perimenopausal symptoms and acknowledged that there were gaps in their training. Time constraints and the stigma surrounding menopause further hindered consultations.

**Conclusion:**

Addressing mental health symptoms during perimenopause requires a proactive and informed approach in primary care. Improved GP training on menopause, coupled with patient education to increase awareness and confidence, could improve consultations and the management of mental health symptoms experienced during perimenopause.

## How this fits in

Mood changes are a recognised symptom of perimenopause, for which hormone replacement therapy is considered a first-line treatment. Recent studies have found that mental health symptoms are overlooked in menopause care, which is mostly delivered by primary care practitioners. This study highlighted some key barriers to identifying perimenopause in women presenting with mental health symptoms; it also proposes important changes clinicians can make to their consultations to address this, along with further research that would be beneficial.

## Introduction

Perimenopause is the late reproductive stage of a woman’s life, during which ovarian reserve is low and causes hormonal fluctuations. It is recognised as starting with changes to the menstrual cycle and finishing 1 year after the last menstrual period.^1^ This hormonal transition can cause multiple symptoms; these last for an average of 4 years, but duration can be up to 11 years for some women.^2^

The mental health symptoms associated with perimenopause are often overlooked by both women and clinicians, despite an increased risk of mental health changes during this time.^3^ Research found that women were two to four times more likely to experience a major depressive episode in perimenopause than in premenopause,^4^ and that women with no premenopausal history of anxiety were more likely to experience this during perimenopause.^5^ However, women may not be aware that mood changes can be associated with the menopause. Women consulting with a GP for mental health support may also be unaware of other perimenopause-related symptoms and, therefore, may not mention these during their consultation.

Mental health symptoms are a common reason for presentation in primary care and may be attributed to other stressors in midlife, with no consideration given to the impact of perimenopause.^6^

There is also a lack of comprehensive NHS guidance and adequate GP training in this area, which may further impede diagnosis. The National Institute for Health and Care Excellence does not mention perimenopause as a contributory factor for anxiety and relies on the practitioner identifying the woman’s menopausal status to consider this in depression; as such, perimenopause may not be considered by a GP seeing a patient with mood changes at their primary presentation, particularly if the patient themselves does not mention other menopausal symptoms in consultation.^7,8^ Despite the majority of menopause care being conducted in primary care, menopause does not form a significant part of GP training, and recent studies have highlighted the effect this has on GPs’ confidence in managing menopause consultations.^6,9^

There is little qualitative research exploring patients’ understanding of mood changes in perimenopause and their experiences of presenting to primary care with mental health symptoms when they are within the perimenopausal age range. There is also limited research exploring GPs’ understanding of mood changes during perimenopause and how this may impact mental health-related consultations in women in the perimenopausal age range.

### Aims

This study aimed to explore the experiences of clinical consultations in:

- patients who were in the perimenopausal age range presenting with mental health symptoms, and
- GPs providing care to such patients.

## Method

A qualitative, semi-structured interview study was conducted with patients and GPs in one integrated care system (ICS) that had a population of ~1.1 million^10^ in the south-west region of the UK. A patient and public involvement (PPI) group comprising six women from diverse ethnic and sociodemographic groups with experience of perimenopause symptoms was established prior to recruitment. Group members ensured the topic guide was appropriate and identified effective recruitment strategies.

The Standards for Reporting Qualitative Research framework^11^ were adhered to.

### Participants, setting, and recruitment

Eligible patient participants were women aged 45–55 years who had presented to their GP in the previous 6 months with a mental health symptom, such as low mood or anxiety. They were recruited from six GP surgeries through the National Institute for Health and Care Research (NIHR) Clinical Research Network (CRN) from Bristol, North Somerset and South Gloucestershire (BNSSG) ICS. Women who met the eligibility criteria were sent a text message containing information on the study by participating practices. Large local employers and community groups also shared study information with their mailing lists. Women were excluded if they were:

- post-menopausal (last menstrual period >1 year previously),
- on hormone replacement therapy (HRT) at the time of their consultation,
- being seen by secondary care for mental health issues, or
- could not speak English adequately enough to participate in an interview.

Patients were directed to the study website that contained the patient information leaflet and consent form. Interested patients completed an online form to provide their demographic data (age, ethnicity, postcode) and contact details. Potential participants were emailed to confirm their eligibility and discuss the study; this was also a point at which any questions they had could be answered. An online video call for the interview was arranged and a £25 shopping voucher was provided for participation.

GPs in the ICS were recruited through practice emails and local GP social media groups; they were directed to the project website and asked to complete an expression of interest form, along with details of their surgery and any specific menopause training they had undertaken. They were subsequently emailed to arrange an online interview and also received a £25 shopping voucher for participation.

### Data collection

The topic guides (Supplementary Box 1) were developed iteratively. Questions were generated by one researcher with feedback from the project team and the PPI group. The patient topic guide included questions on the reason for their visit, the context of their symptoms, what they were asked in the consultation, and the treatment that was offered to them. There were also questions about the patients’ awareness and knowledge of perimenopause, and whether they felt they were experiencing any perimenopausal symptoms. The GP topic guide focused on their approach to mental health consultations, experience with mental health presentations in perimenopause, and any prior menopause training that had been undertaken.

The interviews were conducted between February 2023 and August 2023. All participants gave informed consent prior to the interview using an online form in REDCap. Interviews lasted 30–60 minutes, were audio- and video-recorded and transcribed verbatim, then checked for accuracy before coding was undertaken.

### Data analysis

Transcripts were thematically analysed in NVivo (version 12); initial deductive coding was based on the topic guide and further coding was generated inductively.^12^ Three of the researchers each read the transcripts of four patient interviews and four GP interviews, then met to discuss broad initial themes.

One of the researchers generated an initial coding framework after two readings of all transcripts; another subsequently coded a subset of both patient and GP interviews and generated an independent coding framework. Both of these researchers met to agree where themes overlapped or could be unified, or to decide whether a new theme or sub-theme was required. The researcher who had generated the initial coding framework then completed the remaining coding using the unified coding framework. Comparisons were made to identify overlapping themes between GPs and patients. Recruitment continued until no new themes were identified.^13^

### Reflexivity

The two researchers who met to agree the themes are both GPs with a special interest in menopause care and work in the same geographic area as the study participants. One conducted the interviews, which allowed for a clinical understanding of both the patient journeys and GP experiences. An additional researcher, who is experienced in qualitative research in women’s health, provided guidance on methodology and analysis, along with an in-depth review of themes and sub-themes in relation to the data. A further researcher is a professor in primary care, with expertise in mental health research in a primary care setting.

## Results

### Participant characteristics

Participant characteristics are shown in Table 1.

**Table 1.** Participant characteristics

Participant type and characteristic	*n* ^a^
**Patients, *n* = 18**	
Age, years, mean	48
Education, *n* = 18	
Secondary/college/vocational	8
Graduate/postgraduate	10
Ethnicity, *n* = 18	
White	16
Asian	2
IMD decile, *n* = 18	
1–5	8
6–10	10
Perimenopausal symptoms mentioned in interview	
Vasomotor symptoms, *n* = 18	12
Started HRT, *n* = 18	4
**GPs, *n* = 11**	
Gender, *n* = 11	
Man	5
Woman	6
Career stage, *n* = 11	
GP registrar	3
≤5 years of qualification	4
>5 years since qualification	4
Practice IMD, *n* = 11	
1–5	6
6–10	4

^a^Unless otherwise specified.

HRT = hormone replacement therapy; IMD = Index of Multiple Deprivation;

#### Patients

Of 54 patients who expressed interest in participation, 34 were eligible. The most common reason for being ineligible was being on HRT at the time of the consultation. Eighteen of the 34 eligible women proceeded to interview, following receipt of the study information leaflet and consent form; 16 did not respond to their invitation for interview.

The mean age of patient participants was 48 years, and just over half (*n* = 10) had a graduate or postgraduate degree. Eight participants were in IMD deciles 1–5, which indicated greater deprivation. Most patients reported other perimenopausal symptoms, the majority of which were vasomotor symptoms. Subsequent to their consultation, but prior to interview, four women had started taking HRT; these women were included in the analysis as it was considered important to ensure that women whose mood changes had been identified as linked to perimenopause during or after their consultation were not excluded.

#### GPs

In total, 11 GPs made contact regarding the study and all proceeded to interview. There was a relatively even distribution of gender and level of practice deprivation. Two GPs were from the same practice.

### Themes

Themes and sub-themes for patients and GPs are detailed in Supplementary Tables 1 and 2. The data were organised into four shared themes — uncertainty, consultation context, management, awareness — across GP and patient interviews. Five unique themes were identified for patients:

- consultation experience,
- personal context,
- impact,
- medical history, and
- experience with hormones.

Five unique themes were also identified for GPs:

- approach to consultations,
- presentations,
- shared decision making,
- agenda setting, and
- clinical experience.

**Figure 1.**
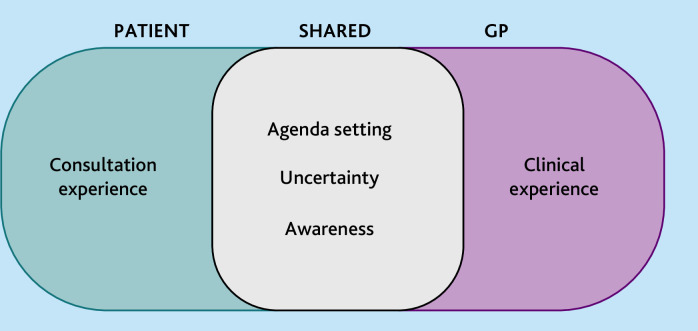
Presented themes and sub-themes for GPs and patients.

The most prominent shared and unique themes that elicited the richest data (Figure 1) will be discussed. Agenda setting was considered to be a theme for GPs due to the substantial amount of data collected; though patients also discussed agenda setting, this was classified as a sub-theme under ‘consultation experience’ as less emphasis was placed on this by patients.

#### Agenda setting

Both patients and GPs described patient-led agenda setting in consultations. Though GPs described this as a distinct component of the consultation, patients discussed this in the context of their overall consultation experience. Many patients reported being asked to describe why they had come, or what they wanted, at the beginning of the consultation. Some patients perceived this to be a helpful shortcut to getting what they needed; others felt they needed more support from the GP to help identify what they needed:

*‘I said “This is what I want” because, invariably, most doctor’s appointments are like*, *“What can I do for you?”. So I was like, “Well this is the issue, this is the problem, I think this* [HRT] *might be the solution”*, *and he said “Yeah I agree, I think we should try you on a low dose”.’* Patient 16

*‘She said “What do you need from me?” And I’m like, “I don’t know at the moment, I don’t know what I want, I don’t know what I need, but I know I need to do something”.’* Patient 18

GPs agreed with this and perceived their consultations to be patient led; they reported using questions to establish the patient’s expectations and to define what would be discussed as early as possible during the consultation. This was seen as important for meeting patient expectations and being efficient:

*‘We generally don’t have that much time do we? So you … and I think when you’ve been in practice quite a long time you get good at getting to the shortcut and finding out what they actually … want.’* GP 10, male, GP partner

Many GPs reported that they would not routinely enquire about perimenopausal symptoms when a patient presented with mental health symptoms, even for patients in the perimenopausal age range. Most reported utilising a standardised approach to a patient presenting with a mental health symptom that was initially very open and patient led, and led onto routine mental health questions regarding risk of self-harm and suicide; this rarely changed based on the patient’s age or sex:

*‘I guess I’ve seen people consider menopause as one of the causes, or I’ve seen patients just bring it up themselves. But I guess I wouldn’t say that I think about it a lot when I see people. I don’t look at their age and think “Could it be menopause?”.’* GP 8, female, GP registrar

*‘In that age group, it wouldn’t be my most immediate thought that it would be perimenopausal without the patient mentioning that they think they have menopausal symptoms.’* GP 2, male, newly qualified GP (NQGP)

Though many GPs described routinely asking about some specific physical symptoms associated with mental health — including sleep, appetite, and palpitations — many stated that they would be unlikely to explore key perimenopausal symptoms, such as a change in menstruation or hot flushes, even for patients in the perimenopausal age range:

*‘I think probably, honestly, I probably haven’t specifically, unless there’s something that they say that suggests to me … “I’m feeling really sweaty”. They might volunteer it. I don’t think I’ve put it as part of my checks that you do at the end, like your closed question bit … so I haven’t really been doing that, and then only if it comes up I suppose.’* GP 10, male, GP partner

In contrast, however, some GPs — particularly those with an interest in women’s health — did report asking patients about their periods and felt this also informed them about other mood disturbances linked to hormonal fluctuations, such as premenstrual syndrome and premenstrual dysphoric disorder:

*‘Sometimes they’re just presenting with the mental health side of things, and even you asking where they’re at with their periods can help them think “Oh, could that be related to what’s going on right now?”. Sometimes it’s the first time they’ve made that potential link.’* GP 1, female, salaried GP

#### Consultation experience

Some patients reported being aware that their symptoms could be linked to perimenopause, but experienced other barriers to addressing this during their consultation. One patient mentioned embarrassment:

*‘I do, I get night sweats, I get palpitations and I don’t think that’s connected to the other things. I just don’t sometimes feel in control. At the same time, despite all my health problems, I actually feel embarrassed to ask.’* Patient 11

Another felt that raising the issue was breaching the time allocated for the consultation:

*‘I did go into that consultation thinking “I’m not really allowed to talk about it”, so I did, but I was quite conscious of “I don’t want to take too much time doing this”.’* Patient 17

Even when a patient did feel confident to discuss perimenopause with the GP, this was not necessarily a facilitator to receive the support they required. One patient who had discussed perimenopause with her GP had her concerns dismissed:

*‘The doctor was rushed, and said “The thing is it’s just not … there is no actual evidence that this is hormonal at all, it’s very likely to be depression, especially as the fact you’ve got a history of it”, and I said “But does my age not come into this?”. And I found out my family history is menopause quite young, and he said “I don’t think that’s got any bearing on it, and we’re getting a lot of this”, he said. “There’s a lot of hysteria about it, and the media hasn’t necessarily helped.”.’* Patient 7

However, one patient whose GP did initiate a conversation about perimenopause found it helpful and, in turn, it informed their understanding on the menopause transition:

*‘When I went to the doctor recently* [they] *said “Oh well, you can have things happening from the age of 40.” I didn’t realise that, I thought that was just in the, maybe, couple of years leading up to the point where your period is due to stop is when you start noticing stuff.’* Patient 6

#### Uncertainty

Patients described finding it difficult to know whether their mental health symptoms could be influenced by hormonal fluctuations in perimenopause. Most patients initially attributed their symptoms to social circumstances and recent life events. However, many felt that hormones could be an additional factor in their mental health symptoms, and that perimenopause created instability or lowered their resilience for dealing with the normal stressors in their everyday lives. Some patients mentioned that they were either using contraception methods, or had had procedures that affected their periods and, therefore, had been unable to tell whether there had been changes to their menstrual cycle. This created further confusion regarding their menopausal stage and whether it played a part in their mental health symptoms:

*‘But I do think that was wrapped up with probably I’d moved country, I’d changed job, I probably … my relationship wasn’t great, so probably* [I] *was burnt out at some point. But I think it’s almost, if you’re informed, it’s almost a natural question when you start to hit your mid-to-late 40s: “Is this something more, is it perimenopause?”.’* Patient 10

*‘So in my head there was a more obvious reason that these things were happening, and I hadn’t really joined the dots if there are dots to be joined, I don’t know.’* Patient 3

GPs noted the difficulty in diagnosing perimenopause in patients presenting with mental health symptoms, particularly for those who had other complex physical conditions and previous mental ill health. Many GPs acknowledged the uncertainty provoked by these consultations and how attaining a balance or ‘middle ground’ without a definitive diagnosis was difficult for both themselves and the patient:

*‘It’s much easier if somebody comes in and says “I’m having awful hot flushes and night sweats, brain fog, and my mood isn’t brilliant”… rather than somebody who comes in and has a textbook presentation of low-level depression or anxiety, and then they say “Oh I wonder if it might be my hormones”. I’m like, “I don’t even know, maybe, I’m not sure,” and then what do we do about it?’* GP 3, male, NQGP

*‘Yeah, depends on … if their presentation is more mental then it will be a mixed approach where lots of factors tie into it. If it’s very much a more menopause kind of thing then the menopause* [is the] *focus, rather than going down a mental health line. But often there’s in-between, which is tricky.’* GP 6, male, NQGP

GPs found it particularly difficult to hold this middle ground when patients felt their symptoms were secondary to hormonal changes, but had a more complex mental health history or other strong risk factors for a primary mental health diagnosis:

*‘But it’s difficult because you get a bit of pushback sometimes to the suggestion that particularly if someone has come in with that [perimenopause] in their mind, you tend to get a pushback if you then try and explore other factors, and sometimes people feel as if you’re dismissing.’* GP 7, female, salaried GP

#### Awareness of perimenopause

Both patients and GPs commented on increasing awareness of perimenopause. Though GPs attributed this mainly to media coverage, patients discussed attending employer workshops and increased awareness due to the experiences of friends and family:

*‘We would discuss it quite a lot at work, and we felt comfortable enough to do that.’* Patient 8

*‘I discussed it with some friends, and some had already been through it, are a bit older than me and stuff, and yeah they said “It sounds like you’ve got perimenopause”, and I start to learn a bit about it, then realise what it was, yeah.’* Patient 9

However, many GPs perceived significant socioeconomic and cultural differences in patients’ awareness of perimenopause. They described how patients from predominantly White, affluent areas were more likely to have greater awareness and set the agenda of their consultation to access support for perimenopausal symptoms, whereas those from more deprived backgrounds might not even recognise their symptoms as being secondary to perimenopause:

*‘I think our area it’s traditionally quite deprived. I think we’re in the bottom 15% of, in terms of deprivation in* [city] *with some of our wards and stuff. So I think the level of education of some of our patients is quite relatively low, and that’s not an insult it’s just a fact. So I think some of them don’t really recognise when* [there are] *symptoms* [of perimenopause] *that are happening to them.’* GP 10, male, GP partner

*‘When I was in* [town] *we had more people of colour, people from different cultures, so I noticed people who were from different cultures might be a bit more hesitant to talk about the menopause.’* GP 5, female, GP trainee

*‘So in one extreme, you might have somebody who’s watched all the Davina documentaries and really* [laughs] *this is definitely what’s going on, and then might be blinkered to other things. Or you might, at the other extreme, well not extreme, but you might also have somebody who goes “Well I’ve battled depression and anxiety all my life, and I know this pattern, and this feels the same”.’*GP 1, female, salaried GP

One patient mentioned that their GP had been the first to mention earlier onset of symptoms. Other patients discussed their lack of awareness of perimenopause until experiencing symptoms themselves, or only finding out quite late in life:

*‘One of my nephews he went to* [country] *as a doctor, and he was making the joke of his auntie, my sister in law, that “Oh they’re going through menopause”. So I ask my son I say “What is menopause?”. And he explain to me, and then now I start realising.’* Patient 13

*‘I didn’t really know much about it before. I just thought one day you stopped and that’s it, I didn’t really know there’s a build-up to it until I started having that experience for myself.’* Patient 9

Personal experience could also influence GP awareness and subsequent interest in perimenopause symptoms, as well as awareness of mental health issues in perimenopause:

*‘I literally had a conversation with a close friend last night, she’s 45 I think, and just her story, I was just like, “I think I’ve heard this story before”. So it’s more from that, that I’ve maybe gained more of an interest about it, because my peer group, and my friends, my family and things are all getting to that age.’* GP 10, male, GP partner

*‘One of my best friend’s mums, when she was going into her perimenopause, had a dip in her mental health, and I remember talking to her about that before starting salaried life, and she was saying “You should always think about mental health stuff associated with menopause for women who are my age, because my GP never really linked the two of them together”.’*GP 3, male, NQGP

#### Clinical experience

All GPs commented on a lack of training on perimenopause and menopuaseduring medical school, which affected confidence managing perimenopause. However, most had participated in some — albeit limited — during their GP training. This was particularly the case for NQGPs, many of whom had completed short modules or lectures, while others with a special interest had completed courses. GPs who had a doctor with a special interest in menopause at their practice found it to be a useful way to keep up to date and discuss difficult cases:

*‘We’re very lucky in that one of the GPs in my practice has done additional training, so she’s now a qualified menopause specialist — I can’t remember what the qualification is exactly — and she’s done a few sessions for us on menopause management.’* GP 7, female, salaried GP

*‘I have not become well versed enough in it to be confident to be like, ”Oh right, you’ve got a uterus but you’re still having periods, so we need to give you blah, blah, blah”, and the flowcharts and things.’* GP 3, male, NQGP

The GPs who had not undergone recent or additional training on menopause were aware of the gap in their knowledge and how this affected their confidence in managing perimenopause in consultations:

*‘I feel like I would benefit from more training, because it’s something I haven’t experienced, so I need to know what happens, and what people actually… what patients’ feedback is, what they benefit from.’* GP 8, female, GP trainee

*‘I feel frustrated sometimes when people bring it up, because I know that I’m not going to be as comfortable navigating the rest of the consultation, because I essentially don’t know.’* GP 3, male, NQGP

## Discussion

### Summary

The authors found substantial barriers to identifying perimenopause as a possible cause of mental health symptoms in women of perimenopausal age. Patient-led consultations disadvantaged those who were unaware of the association or hesitant to discuss perimenopause. Both patients and GPs faced uncertainty in linking these symptoms to perimenopause.

Limited time in consultations obstructed comprehensive assessments. The media, friendships, and workplace discussions helped raise awareness among both patients and GPs, but GPs felt this was not equally experienced and that patients from deprived areas and ethnic minority groups were less likely to seek advice about their concerns.

GPs reported training in menopause and HRT being inadequate, and emphasised the value of working with colleagues with expertise in women’s health who could offer support.

### Strengths and limitations

This study explored the consultation from both the patient and GP perspective, which provides more depth to the profession’s understanding of consultation dynamics and could inform a more-holistic response to improving clinical care for this patient group.

Patients and GPs were recruited from a single ICS in the south-west of England, which meant there was a shared landscape in which the consultations were taking place. This meant the experiences of GPs and patients were influenced by the same service structure and availability. However, this limits the generalisability of the study outside this population. The fact that the interviewer worked in the same geographic area as the interviewees could have unconsciously influenced the depth and direction of questioning. All participants were recruited from areas with varying degrees of deprivation; however, the patient sample had limited ethnic diversity, despite specific targeted recruitment drives through local community groups.

Patients were aware of the topic of interest at recruitment, and this may have attracted those who had pre-existing concerns regarding perimenopause to the study.

### Comparison with existing literature

This research highlights the importance of the GPs’ role in facilitating consultations, particularly where awareness of some symptoms may be low, or patients may feel inhibited or stigmatised when bringing up symptoms. A clear distinction should be made between patient-centred care and patient-led care. Miller and Fritz have made the point that patient-led care can lead to missed diagnoses and widening of health inequalities when the GPs do not use their skills to uncover cues and symptoms and guide patients through uncertainty.^14^ GPs prioritising the patient agenda without discussing associated symptoms can prematurely narrow further information gathering and lead to misattribution of symptoms. Failure to elicit the connection between mental health and perimenopause can lead to misdiagnosis or inappropriate treatment. Assigning perimenopause-related mood changes to primary psychiatric disorders could lead to the prescription of antidepressants when HRT might be more appropriate and perhaps more effective;^15^ this is particularly important when patients may be experiencing hot flushes, vaginal dryness, and poor sleep — all of which can have an impact on mood.^16,17^

Uncertainty and lack of confidence were obstacles to addressing perimenopause in the consultation for some patients. A similar phenomenon has been noted in sexual health settings: patients reported feeling unable to initiate a conversation due to embarrassment, but said they wanted to be asked.^18^ A recent qualitative review of women’s experiences of menopause found this is compounded by lack of time in GP appointments and limited access to specialist menopause clinics.^19^ This is significant given that one of the strongest predictors of HRT use is recommendation by a primary care provider.^20^ These obstacles may be particularly difficult to overcome in underserved communities; recent research has highlighted the inverse relationship between HRT prescribing rates and deprivation deciles.^21^

The results reported here support findings that stigma and normalisation of menopause still pose significant barriers to seeking support for symptoms.^6^ As menopause is a natural life stage, some patients may feel that this does not entitle them to medical support.^6^ A recent study in the UK found that mental health was particularly overlooked in menopause care, despite most patients reporting that menopause symptoms affected their mental health;^3^ the findings presented here suggest a similar situation for patients with mental health presentations as they, in turn, were not asked about perimenopause symptoms.

GPs’ reflections on their lack of standardised training echo those of a recent survey of UK GPs, which reported that 77.5% of responders felt that medical training on menopause needed to be improved.^22^

### Implications for research and/or practice

**Figure 2.**
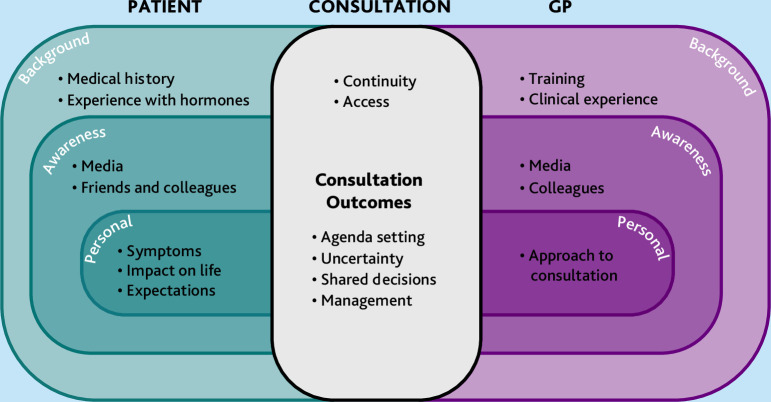
In-depth representation of selected themes and subthemes for GPs and patients

Figure 2 illustrates the complex interplay between patients, GPs, and the consultation process, showing how different layers of background, awareness, and personal context shape the interaction. These pre-existing factors can either facilitate or inhibit the identification and management of perimenopausal symptoms, especially those that affect mental health. To improve diagnosis and care, interventions should address both patients’ and GPs’ needs, with tailored approaches targeting different layers.

Increased GP awareness of the mood symptoms that are associated with perimenopause could lead to a more proactive approach to management. Questions about menopausal symptoms could be included when a patient of perimenopausal age attends for a routine smear test and further information could be provided, with follow-up consultations for women who require further support. Smear tests provide a routine opportunity to speak to women who may not have considered perimenopause before and would not approach their GP for advice.

Moving to longer appointment times would undoubtedly support consultations being more thorough. However, even including a simple question such as ‘Have you noticed any change in your periods?’ for women presenting with mental health symptoms in the perimenopausal age range may provide a space for patients to either reflect on their symptoms or air any pre-existing concerns.

GPs working in deprived or multi-ethnic areas could play a particularly important role in raising awareness and providing education on perimenopause and how it can affect mental health. Menopause initiatives in primary care, such as group consultations and webinars, might have a positive impact on women’s understanding of the menopause transition and their confidence in seeking further support.

The management of mental health symptoms during perimenopause in primary care requires a more proactive approach from GPs. Currently, enquiries about perimenopausal symptoms are sparse, which means many women, especially those who are unaware of the connection between menopause and mental health, may not have their symptoms adequately addressed. The lack of GP training on menopause exacerbates these challenges.

Addressing this gap in medical education and increasing awareness of perimenopausal symptoms during consultations could help to improve care. GPs would benefit from standardised training to increase awareness of mental health presentations in the perimenopausal age range and should be encouraged to address this directly in consultations. Further research is needed to understand the most effective way of providing a comprehensive menopause education at both medical school and in GP specialist training.

## Data

All data requests should be submitted to the corresponding author for consideration. Access to anonymised data may be granted following review via the University of Bristol Research Data Storage Facility.
